# Reward-Modulated Hebbian Plasticity as Leverage for Partially Embodied Control in Compliant Robotics

**DOI:** 10.3389/fnbot.2015.00009

**Published:** 2015-08-17

**Authors:** Jeroen Burms, Ken Caluwaerts, Joni Dambre

**Affiliations:** ^1^Computing Systems Laboratory (Reservoir Team), Electronics and Information Systems Department (ELIS), Ghent University, Ghent, Belgium; ^2^Intelligent Robotics Group, NASA Ames Research Center, Oak Ridge Associated Universities, Moffett Field, CA, USA

**Keywords:** compliant robotics, Hebbian plasticity, morphological computation, recurrent neural networks, tensegrity

## Abstract

In embodied computation (or morphological computation), part of the complexity of motor control is offloaded to the body dynamics. We demonstrate that a simple Hebbian-like learning rule can be used to train systems with (partial) embodiment, and can be extended outside of the scope of traditional neural networks. To this end, we apply the learning rule to optimize the connection weights of recurrent neural networks with different topologies and for various tasks. We then apply this learning rule to a simulated compliant tensegrity robot by optimizing static feedback controllers that directly exploit the dynamics of the robot body. This leads to partially embodied controllers, i.e., hybrid controllers that naturally integrate the computations that are performed by the robot body into a neural network architecture. Our results demonstrate the universal applicability of reward-modulated Hebbian learning. Furthermore, they demonstrate the robustness of systems trained with the learning rule. This study strengthens our belief that compliant robots should or can be seen as computational units, instead of dumb hardware that needs a complex controller. This link between compliant robotics and neural networks is also the main reason for our search for simple universal learning rules for both neural networks and robotics.

## 1 Introduction

Hebbian theory has been around for over half a century (Hebb, 1949), but it still sparks the interest of today’s researchers. Small changes to the basic correlation learning rule result in various well-known algorithms, such as principal (Oja, 1982; Sanger, 1989) or independent component (Hyvrinen and Oja, 2000; Clopath et al., 2008) extractor networks. The basic rule is biologically plausible as are some of its variations (Mazzoni et al., 1991; Loewenstein and Seung, 2006). Whereas all these approaches belong to the general category of unsupervised learning, *reward modulated Hebbian* (RMH) learning is similar to reinforcement learning in that it can be used to tune a neural system to solve a specific task without the need to know the desired output signals at the neural level (Fiete and Seung, 2006; Legenstein et al., 2010; Hoerzer et al., 2012; Soltoggio and Steil, 2013; Soltoggio et al., 2013). When using RMH learning in a robotics context, a reward can be computed, e.g., by comparing the sensory inputs with the desired observations. The use of RMH learning for optimizing robot motor control has several additional advantages. First, the basic learning rule is simple. There is no need for complex mathematical operations and it can therefore be efficiently implemented on various platforms in hardware and software. Second, it allows for a distributed implementation: a central unit can be responsible for a global reward, which can then be broadcast to the learning units of local controllers. Finally, RMH learning is an online learning approach. If the reward mechanism remains active, the controller can adapt to changes in the robot morphology or dynamics, e.g., due to wear or damage.

Class one tensegrity structures (Skelton and de Oliveira, 2009; Caluwaerts et al., 2014) consist of compression members held together by tension members in such a way that compression members are never directly connected. In robotics, these are typically a set of rods, interconnected by tension elements (springs or cables) between the rods’ endpoints. These structures can serve as compliant robot bodies, by allowing some or all of the tension elements to be actuated. This results in flexible pin-jointed structures that make efficient use of materials, and are both extremely robust and lightweight (Caluwaerts et al., 2014). Tensegrities have also been researched from various other perspectives, from architecture and art (Snelson, 1965) to mathematics (Connelly and Back, 1998) and even biology (Ingber, 1997).

In previous work (Caluwaerts et al., 2012), we demonstrated that the motor control of a tensegrity robot can be drastically simplified by using its body as a computational resource. This approach originated from the concepts of *physical reservoir computing* (Verstraeten et al., 2007) and *morphological computation* (Pfeifer and Bongard, 2007), both of which treat the use of physical systems or *bodies* as a computational resource in so-called embodied computation. In Caluwaerts et al. (2012), we mainly focused on approximating motor signals through a single layer linear neural network acting as a feedback controller. The flexibility and lack of joints of our tensegrity robot allowed for simple learning rules, as the risk of failure due to mechanical stress or hard constraints was minimal. As a consequence, the feedback weights were learned by applying online supervised learning rules to approximate the target motor signals, among which was a supervised version of RMH learning.

Various forms of RMH learning rules have already been extensively studied in the context of both spiking (Fiete and Seung, 2006; Izhikevich, 2007; Legenstein et al., 2008) and rate-based (Loewenstein and Seung, 2006; Loewenstein, 2008; Soltoggio and Steil, 2013; Soltoggio et al., 2013) neural networks. The learning rule we handle uses noise as an exploratory term, similar to Legenstein et al. (2010), and can be shown to approximate gradient descend (Fiete and Seung, 2006). In this paper, we show that the RMH learning rule can be extended to systems exhibiting partial embodiment, i.e., agents that actively see and use their body as a computational resource. In these partially embodied systems, the computations that are performed by the body are naturally integrated into the controller architecture. We use the term “partially” to make the distinction with full embodiment, where agents do not need a controller, and with “trivial” embodiment, where little to no computations are offloaded to the body.

We first consider various analog recurrent neural network tasks and setups. Second, we will demonstrate that the RMH learning rule can be carried over beyond the scope of neural networks. We train the linear feedback weights of a secondary controller in a two-level control hierarchy for end-effector control in a highly compliant, simulated class one tensegrity robot. The primary controller – a simple feed forward kinematic controller – generates control signals derived from a very rough static inverse model of the relationship between end-effector positions and actuator signals. The secondary embodied controller, consisting only of the robot body and linear feedback weights, handles the dynamics, i.e., it tunes the primary control signals to result in smooth and stable trajectories. In this task, only the desired end-effector trajectories are known, not the control signals required to generate them.

Thus far, in physical reservoir computing, embodied or morphological computation has always been exploited using supervised learning techniques. This implies that the target motor signals have to be known (e.g., determined using evolutionary techniques as in our own previous work) and fixed. However, in compliant robotics, it is important that the controller can adapt to variability in its surroundings as well as to changes of its own body. From this point of view, a reward-modulated approach is much more suitable. We demonstrate how these systems can be effectively trained in an entirely online manner.

## 2 Methods

In this section, we introduce the basic learning rule used throughout the paper in the context of neural networks, and we discuss additional changes to the rule to make it more suitable for the targeted application in partially embodied control of a tensegrity robot.

Throughout this work, we will employ the term *observations* instead of state or network activity, in order to emphasize that the learning rule is also applied in a more general context than neural networks.

### 2.1 Hebbian learning in analog recurrent neural networks

Hebbian plasticity is a biologically plausible learning methodology for neural networks. A learning rule is called Hebbian if it modifies the weights between a set of presynaptic neurons ***x*** and postsynaptic neurons ***y*** as a function of their joint activity. Although Hebb (1949) did not provide a precise mathematical formulation of his postulate, a relatively general form can be written as:

(1) $$\Delta {\text{W}}_{\text{Hebb}}=f\left(\text{X},\text{Y}\right).$$

Note that we have used capital ***X*** and ***Y***^1^ to indicate that the weight updates in the learning rule can depend on multiple time steps, i.e., the history of the pre- and postsynaptic neuron activations.

To apply Hebbian theory in a reinforcement learning setting, we have to introduce the notion of a reward *r* into the learning rule. Indeed, reinforcement learning aims at making behavior that optimizes the reward more likely to happen. However, learning new behaviors necessitates another tool, namely exploration. We use noise ***z*** injected at the postsynaptic neurons for exploration.

If the exploratory noise causes an improvement in behavior, this will result in a higher reward (and vice versa). A basic learning rule based on this idea is:

(2) $$\Delta \text{W}=r\text{z}{\text{x}}^{T}.$$

Note that the postsynaptic neuron activations ***y*** are only indirectly considered in this weight update: the noise ***z*** can be viewed as a cause for variations in ***y***, and could be computed from the expected and noisy postsynaptic activations.

However, this rule suffers from a number of basic flaws. First, credit is only assigned to the exploratory noise that was inserted in the same time step that the reward was received. For the learning rule to be able to credit both past and present exploration, some efficient notion of memory of the relationship between exploration noise and the presynaptic neuron states needs to be present. This can be achieved by computing the covariance between the exploration and the presynaptic neuron states throughout multiple time steps. To this end, we will apply the rule on a trial-by-trial basis. Second, we note that in its current form, any significant bias of the reward *r* will cause unfavorable results. The solution to this is to predict the reward and subtract this from the obtained reward, resulting in a learning rule of the form:

(3) $$\Delta \text{W}=\alpha \left(r-\bar{r}\right){\text{Z}}^{T}\text{X},$$

in which $\bar{r}$ is the predicted reward and where we have added a learning rate parameter *α*. The matrices ***X*** and ***Z*** contain the presynaptic neuron states and the exploratory noise throughout the trial, respectively.

The predicted reward is sometimes ambiguously referred to as the (short term) average reward. More precisely, it is the average (or expected) reward when noise is present in the system. As we will demonstrate, the average reward is typically highly dependent on the noise level of the system. The learning rule therefore optimizes the expected reward while noise is present in the system (i.e., maxE[r|z]), under the assumption that this also optimizes the performance when the exploration noise is removed (i.e., maxE[r|z=0]).

Although RMH learning is stable in practice, it is possible to constrain the norm of the weights. This can be useful to do for practical reasons. In a robotics application, for example, this would allow for limiting the required feedback gain and thus the required motor power. In the Appendix, we show that in doing so, the resulting learning rule very closely resembles Sanger’s rule (Sanger, 1989).

### 2.2 Decorrelated learning rule for robotics experiments

In partially embodied control, the dynamics of the robot body are used directly as a computational resource. In our RMH learning setup, this is equivalent to replacing part of the neural network by the robot body, which receives inputs from the remaining neurons. The observations, i.e., the sensor readouts, are fed back into the neurons. The training procedure is now heavily constrained, as it can only adapt synaptic weights of the remaining neurons, whereas the part of the network that is replaced by the body remains unchanged. Nonetheless, the RMH learning rule remains applicable, but the observations ***x*** and the noise ***z*** now include the sensor readouts and motor actuation noise, respectively.

Although this situation is similar to RMH learning in neural networks, it differs in the fact that most of the physical state of the robot remains hidden to the observer and the number of observable signals that can be fed into the trainable neural network is relatively small. Furthermore, the dynamics of the observed variables tend to be highly correlated. For example, stiffening the structure typically causes an increase in all sensor values.

The RMH learning relies on the varying influence of exploration noise on the observed variables. As we will show, a common influence (increase or decrease) of the noise on all observed variables reduces the effectiveness of the learning rule. A simple approach to overcome this issue is to decorrelate the observations. In Caluwaerts et al. (2012) (see Appendix), we showed that a decorrelation layer that uses Sanger’s rule (Sanger, 1989) offers a biologically plausible solution for this. In this work, we take a more pragmatic approach and decorrelate ***X*** on a trial-by-trial basis, using the Moore–Penrose pseudoinverse. The resulting learning rule is given by:

(4) $$\Delta \text{W}=\alpha \left(r-\bar{r}\right){\text{Z}}^{T}\text{X}{\left({\text{X}}^{T}\text{X}+\lambda \text{I}\right)}^{-1},$$

with λ acting as a regularization parameter determining the strength of the decorrelation. A slight variant of this rule is:

(5) $$\Delta \text{W}=\alpha H\left(r-\bar{r}\right){\text{Z}}^{T}\text{X}{\left({\text{X}}^{T}\text{X}+\lambda \text{I}\right)}^{-1},$$

where H(⋅) represents the Heaviside step function. In this variant of the learning rule, weight updates only occur when the observed reward is better than expected. This is not strictly necessary, but we found that it slightly improved our results.

This learning rule is similar to ridge regression, the difference being that the algorithm will try to reproduce the noise ***Z*** (instead of a desired output) proportionally to the reward with injected noise, relative to the expected reward. We used a large regularization parameter (λ = 1), which results in only a minor decorrelation of the sensor variables, yet is enough to allow for efficient learning. A high-regularization parameter allows for simple covariance estimators, in case a more biologically plausible version of the rule is desired.

## 3 Experiments

### 3.1 Neural network experimental setup

Before presenting our results in a robotics context, we first study RMH plasticity in discrete time recurrent neural networks with hyperbolic tangent activation functions. They receive input, which we denote ***U*** and a readout function provides observations of the network state. Additionally, exploration noise ***Z*** is injected into the network. The network update equation is given by:

(6) $$\text{x}\left[k+1\right]=\text{tanh}\left(\text{Wx}\left[k\right]+{\text{W}}_{\text{in}}\text{u}\left[k+1\right]+\text{z}\left[k+1\right]\right).$$

For each of our neural network experiments, the network is initialized according to the reservoir computing approach (Verstraeten et al., 2007). This implies that we initialized the weights randomly (i.i.d. standard normally distributed samples) and then tune the network dynamics to a useful regime. This is achieved by rescaling the weight matrix ***W*** such that its spectral radius – the largest amongst the absolute values of its eigenvalues – is such that the learning converges. For the experiments we will describe, we obtained good performance for initial spectral radii in [0.80, 1.2], i.e., stable or almost stable networks. This differs from related approaches, such as Legenstein et al. (2010) and Hoerzer et al. (2012), where initially chaotic networks are used. The input weight matrix ***W***_in_ was sparsely initialized (20% non-zero elements) with i.i.d. normally distributed values with standard deviation (SD) 0.05. All networks contained 100 neurons.

**Figure 1:**
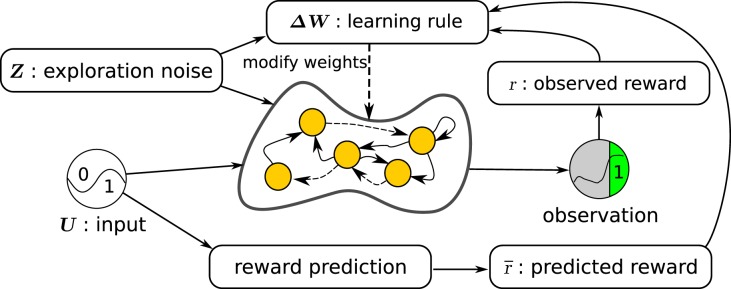
**Overview of the learning setup for recurrent neural networks**. The initially random recurrent neural network receives the inputs ***U*** and the exploration noise ***Z***. The state of the postsynaptic neurons is computed by applying the hyperbolic tangent function to the sum of the inputs, the noise, and the weighted sum of the presynaptic neurons. Observations are made of the state of the network and after every trial (fixed number of time steps), a reward is computed, based on the observations made during the last trial. In parallel, a simple reward prediction network predicts the expected reward for the given input. The learning rule then updates the weights between the presynaptic and postsynaptic neurons, by using the reward, the expected reward, the exploration noise, and the states of the presynaptic neurons.

Figure 1 shows our learning setup for neural networks. The neural network to be trained is the central element. A reward is provided after a trial based on the network observations throughout that trial. A trial is defined as the number of time steps in which the network tries to perform a task of interest. In parallel to the network, a reward prediction system estimates the expected reward based on the network inputs. The RMH learning rule finally combines information from the network state, exploration noise, reward, and estimated expected reward to compute an update Δ***W*** of the network weights.

### 3.2 Neural network experiments

**Figure 2:**
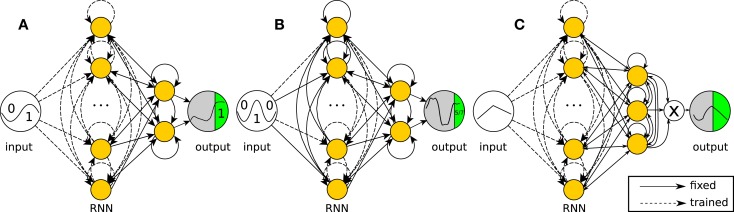
**Network structures for the recurrent neural network tasks**. The networks are simulated in discrete time with hyperbolic tangent neurons (yellow nodes). Full lines are fixed connections, while dashed lines are trained. The reward is evaluated at the output neuron over the green period of time (one reward per trial). **(A)** Task 1 (2-bit delayed XOR): the output equals the sum of two neurons, but only the internal connections can be modified by the algorithm. **(B)** Task 2 (3-bit decoder): the output equals the sum of two neurons as in the XOR task, but now only half of the internal weights can be modified. **(C)** Task 3 (continuous input task): the output equals the product of three neurons. The network has to reproduce the reversed input from the first 5 time steps of each trial.

The networks used for the three tasks described below are shown in Figure 2. They only differ in the way observed outputs are generated and in the subset of weights that can be modified by the learning rule. In the first two networks, two neurons are randomly selected as output-generating neurons, and the output is computed as the sum of their states. The third network has three output-generating neurons and has an output equal to the product of these neurons’ states.

In all networks, the weights to and from the output neurons are fixed and recurrent. This prevents the learning algorithm from generating solutions in which the observation neurons become a pure output layer, which does not influence the state of the rest of the network. In the networks for tasks 1 and 3, all other internal weights are modifiable. In task 2, the training is further restricted by fixing the input weights for half of the remaining neurons. This means that about half of the network is a random recurrent neural network. In a neural or neurorobotics context, we can see this as a rudimentary model for a trainable network interacting with an untrained dynamical system, such as another brain area or a physical body, e.g., the partially embodied control of a robot arm with a neural network.

We purposely chose to have different and unconventional tasks and setups, to display the wide applicability of reward-modulated Hebbian learning. In what follows, we describe the three neural network tasks in more detail. We first consider problems with discrete input spaces. More precisely, we solve the 2-bit delayed XOR problem and a 3-bit decoder task. Our third example has a continuous input space and a more complex readout function.

#### 3.2.1 Task 1: Proof-of-Principle

##### 3.2.1.1 Inputs

The input signal for this task represents a single bit stream. A zero bit is coded as the negative half period of a sine wave, a one bit as the positive half. For each trial, we randomly select one of the four possible 2-bit input sequences.

##### 3.2.1.2 Desired output

The neural network has to compute the so-called 2-bit delayed XOR task, i.e., the exclusive OR function applied to the last two bits of its input stream, represented as binary values. More concretely, the output of the network should be as close as possible to plus one or minus one during the last half of the second bit.

The XOR task is a common test or benchmark, because the patterns are not linearly separable. A linear network cannot obtain optimal performance for all inputs simultaneously. Therefore, this task is a simple test to verify if the learning rule can exploit the non-linear effects of the network. The task also requires the network to remember a specific part of the input, while ignoring inputs that occurred more than one bit length in the past.

##### 3.2.1.3 Neural network structure

The observations are computed by adding the states of two output neurons, which have fixed (i.e., untrained) incoming and outgoing connections. All other weights are trainable.

##### 3.2.1.4 Reward function

Throughout this manuscript, we use different reward functions. The main reasons for this is that some reward functions are more appropriate for a specific task and to show that the learning rule does not depend on a specific reward function. For the results presented for the 2-bit XOR task, we used minus the mean squared hinge loss, as the hinge loss is a more appropriate reward function for a binary classification task:

(7) $$r_{n}^{2\text{bit}}=\frac{-1}{5}\sum_{k=15}^{19}\max{\left(0,\;1-t_{n}\left[k\right]\left(x_{0}^{\text{o}}\left[20n+k\right]+x_{1}^{\text{o}}\left[20n+k\right]\right)\right)}^{2},$$

where *n* indicates the number of the current trial, ***x***^**o**^ are the neurons that generate the observations and *t*_*n*_[*k*] are the desired observations.

##### 3.2.1.5 Prediction of the expected reward

Estimating the expected reward is trivial in the case of a modest number of different inputs. For the results presented here, we averaged the last 50 rewards per input sequence.

#### 3.2.2 Task 2: Partially Embodied Computation

##### 3.2.2.1 Inputs

For this task, the input is the same as for the previous task. For each trial, we now randomly select one of eight possible 3-bit input sequences.

##### 3.2.2.2 Desired outputs

The network now has to act as a 3-bit digital-to-analog decoder, i.e., it has to produce one of eight equidistant analog values in the range [–1, 1], corresponding to the decimal interpretation of the last three encoded bits it received. Similar to the previous task, the desired value has to be present on the output during the second half of the third bit. This task is more complex and non-linear than the previous one and it requires more memory as well.

##### 3.2.2.3 Neural network structure

The output generation is identical to task one. However, this time only half of the internal weights are trainable.

##### 3.2.2.4 Reward function

For the 3-bit decoder task, the reward value *r*^3bit^ is defined as minus the mean squared error of the observations during the last five time steps of a trial:

(8) $$r_{n}^{3\text{bit}}=\frac{-1}{5}\sum_{k=25}^{29}\;{\left(t_{n}\left[k\right]-x_{0}^{\text{o}}\left[30n+k\right]-x_{1}^{\text{o}}\left[30n+k\right]\right)}^{2}.$$

##### 3.2.2.5 Prediction of the expected reward

The rewards were estimated in the same way as in the previous task.

#### 3.2.3 Task 3: Non-Linear Observation Function

**Figure 3:**
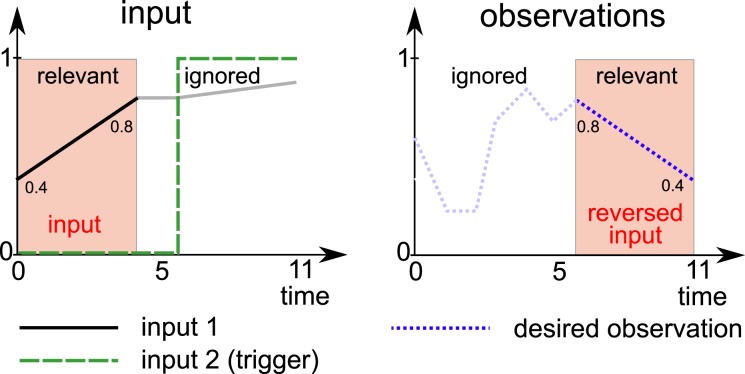
**Overview of task 3: the network has to reproduce part of the first input (black line) in reverse at the end of the trial (dashed blue line)**. More precisely, the first 5 steps of the first input are to be reproduced in reverse at the end of the trial (12 time steps total). The input space consists of a straight line originating and ending in [0, 1]. A second input indicates when the network has to start producing the desired observations.

The task at hand is to reproduce part of the input in reverse after a delay (see Figure 3).

##### 3.2.3.1 Inputs

The network receives two input signals. The first input signal consists of sequences of 12 time steps per trial. The first 5 of these steps form a linear segment between two values, which are sampled with uniform probability form [0, 1] for each trail. The final value is held constant for two more time steps. The final 5 time steps again form a linear segment that is obtained by connecting the last value from the first 5 steps with a third random sample from [0, 1].

Because the trials are fed into the system one by one, it is not clear to the network when a trial starts. The second input, a binary signal, is used to inform the network when it has to generate the desired output.

##### 3.2.3.2 Desired outputs

The network must learn to recall the input during the first 5 steps and reproduce them in reverse order during the last 5 steps of the trial. The system must ignore the remaining 7 input samples.

##### 3.2.3.3 Neural network structure

The observations are computed by multiplying the states of three internal neurons, which have fixed (i.e., untrained) incoming and outgoing connections. All the other weights are trainable.

##### 3.2.3.4 Reward function

The reward function used here is minus the mean absolute error of the observations:

(9) $$r_{n}^{\text{cont}}=\frac{-1}{5}\sum_{k=0}^{4}|u\left[12n+k\right]-\prod_{j=0}^{2}x_{j}^{\text{o}}\left[12n+11-k\right]|.$$

##### 3.2.3.5 Prediction of the expected reward

The expected reward $\bar{r}$ estimates the performance of the system given the noise level *σ*. Furthermore, the reward is input dependent, therefore $\bar{r}$ estimates the following quantity:

(10) $$\bar{r}=E\left[r|\text{u},\sigma \right].$$

Various algorithms can be used to estimate this quantity. We employed the well known recursive least squares algorithm (Kailath et al., 2000) to learn a simple online estimator of this quantity, which we applied to the input sequences ***u*** and the network state ***x*** at the end of a trial.

### 3.3 The tensegrity robot

**Figure 4:**
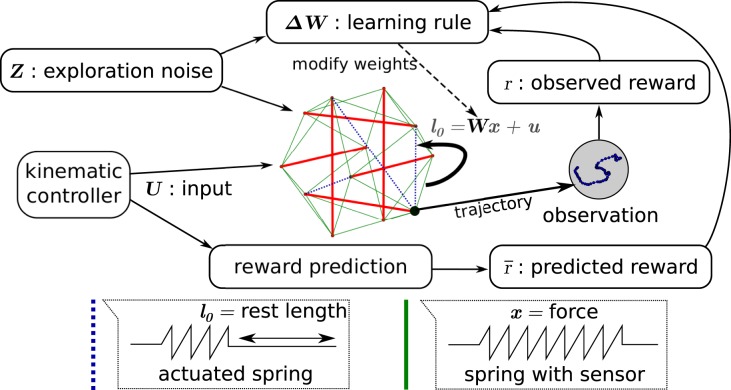
**Overview of the way the learning rule is applied to compliant tensegrity structures**. The setup is similar to the recurrent neural network setup of Figure 1. The neural network has been replaced by the combination of the compliant robot body and the neural linear feedback weights. It now receives input from the kinematic controller. Force sensors on the springs act as presynaptic neurons for the trained weights and the actuator signals correspond to the postsynaptic neurons. The learning rule adapts the feedback weights from the force sensors to the motor signals. The observations used for reward computation are based on the trajectories of an end-effector.

The general setup of our simulated tensegrity robot control problem is shown in Figure 4. It is similar to the neural network setup represented in Figure 1, but the entire recurrent neural network has been replaced with the simulated tensegrity robot. As a result, the only remaining trainable weights are those of a simple linear feedback ***W***, projecting the output to the input.

**Figure 5:**
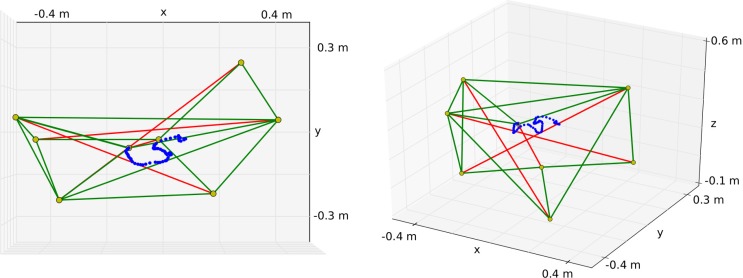
**Tensegrity structure used for the experiments**. The top node of the center rod is used as an end-effector to draw in the XY plane. In this example, the robot draws an “S” as can be seen on the left. The right figure shows another perspective to demonstrate that the reward does not depend on the vertical position.

The tensegrity structure used for our experiments has four struts and is shown in Figure 5. It is based on the standard three strut tensegrity prism (Pugh, 1976) to which a shorter rod has been added that acts as a compliant end-effector. The bottom three nodes of the original prism have been fixed through ball-joints. The resulting structure has seventeen *k* = 20 N⋅m^–1^ springs, 14 of which are actuated (the lengths of the other three bottom springs are fixed). The controller time step was 50 ms and gravity was not modeled.

Instead of observing the state of the neurons, we measure the spring forces:

(11) $$x_{i}=f_{i}$$

(12) $$\;=\max(k(l_{i}-l_{i}^{0}),0).$$

Actuators changing the equilibrium lengths of the springs replace the postsynaptic neurons and motor babbling takes on the role of the exploration noise:

(13) $${\text{l}}^{0}={\text{l}}^{\mathrm{init}}+\text{Wx}+\text{u}+\text{z}.$$

All tensegrity experiments were performed in our tensegrity simulator, which is based on an Euler–Lagrange formulation of the tensegrity dynamics (Skelton and de Oliveira, 2009, chapter 5). For more details on the simulation setup, we refer to Caluwaerts et al. (2012).

### 3.4 Hierarchical end-effector control for the tensegrity robot

The task we consider is writing characters with the top node of the rod suspended in the tensegrity structure. More precisely, the node has to trace letters in a horizontal (XY) plane. The characters were taken from UCI Character Trajectories Data Set (Bache and Lichman, 2013), integrated and then subsampled and rescaled.

The robot is controlled by combining a feed forward kinematic controller and a learned static linear feedback controller. The kinematic controller provides the input signals ***u*** in equation (13). We sampled 100 random spring lengths to create a set of configurations for the kinematic controller. To write a character, the kinematic controller selects a combination of spring lengths that move the end-effector as close as possible to the desired position when the structure is in equilibrium.

The reward function used for the next experiments tries to bring the end-effector close to the desired trajectory:

(14) $$r^{\text{traj}}=\frac{-1}{s}\sum_{k=0}^{s-1}\max\left(∥\text{n}\left[k\right]-\text{c}\left[k\right]∥-0.01,0\right),$$

where *s* is the number of steps required to write the current character, ***c***[*k*] the vector containing the target position at time *k* (relative to the beginning of the trial) and ***n***[*k*] the position in the XY plane of the end-effector at time *k*. This reward function will cause the learning rule to stop improving a feedback controller w.r.t. a point on the trajectory in case the end-effector is within 1 cm of the target position.

### 3.5 Robustness against failures

To demonstrate the robustness of the controllers as well as a more practical application of the learning rule, we simulated various actuator failures. In this case, we used a more realistic feed forward controller. Again starting from a simple kinematic controller, we now optimized the inputs ***u*** in equation (13) at each time step using a basic exploration method. More precisely, a small amount of noise ***z*** is injected at each time step and the change in expected reward is observed. If an improvement of the expected reward is observed after a trial, we reproduce the noise in the feed forward controller ***u*** = ***u****_kin_* + ***u****_expl_* by using Δ***U***_expl_ = ***Z*** when the expected reward (of the trial) improved and Δ***U***_expl_ = 0 when it did not. In the previous equations, ***u****_kin_* refers to the original kinematic controller discussed in the previous section.

In this setup, we now simulate actuator failures by resetting one or more actuators to the original kinematic controller instead of the optimized ones, i.e., ***u*** = ***u***_kin_. At the same time, the kinematic controller is no longer optimized, and instead the learning rule starts learning a set of feedback weights to compensate for the actuator failure.

## 4 Results

### 4.1 Neural network experiments

We first demonstrate the learning rule’s capabilities using the neural network tasks. For these experiments, we always chose the noise level to be *σ* = 0.05 and set the learning rate to be as high as possible, without making the networks diverge. In the context of the basic learning rule [equation (3)] this was *α* = 0.005, whereas the decorrelated version [equation (4)] allowed a much higher learning rate *α* = 0.5. The initial spectral radius was chosen to be 0.95.

**Figure 6:**
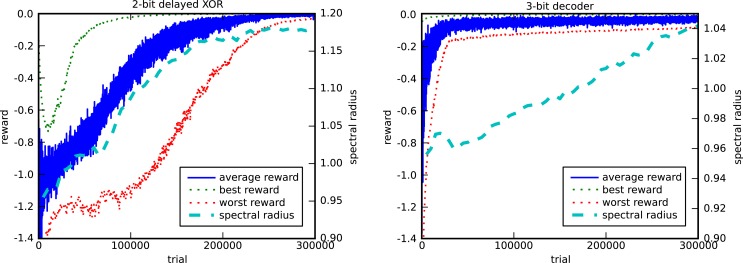
**Evolution of the reward and the spectral radius for the 2-bit delayed XOR task (left) and the 3-bit decoder task (right)**. The plots show the average, best, and worst rewards and the average spectral radius out of 10 runs. Each run has different random initializations and input sequences.

Figure 6 shows the evolution of the reward and the spectral radius for the discrete input tasks (tasks 1 and 2). We performed 10 runs with different random initializations and input sequences. For both tasks, and for every run, we observe that the network indeed learns to solve the task almost perfectly, as the rewards converge to their maximal value of 0.

These experiments also show how the spectral radius evolves as the average reward increases. In the case of the 2-bit delayed XOR task, every run of the algorithm for different initial random weights resulted in a final spectral radius of approximately 1.2, which indicates that the learning rule tunes the memory of the network. In the case of task 2, the learning rule is only allowed to modify half of the internal weights of the recurrent neural network. The network structure can be considered as a model for partially embodied computation, i.e., we replace part of the original trainable network by a fixed one, which acts as a dummy for a physical body. Nonetheless, the learning rule manages to tune the network dynamics to have a spectral radius close to 1.05 after 300,000 trials, which eventually converges to 1.10 (not shown), thus exhibiting the necessary memory.

**Figure 7:**
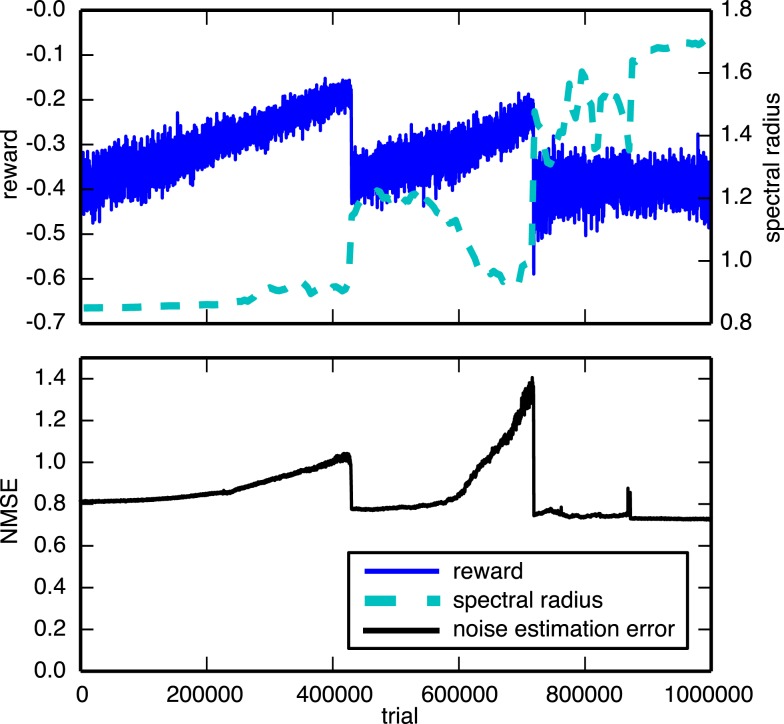
**Evolution of the reward and spectral radius (top) and the noise estimation NMSE (bottom) for the 3-bit decoder task, using a learning rule that uses an estimate of the exploration noise instead of the real noise**. The network consisted of 300 neurons, instead of 100, and was trained over a longer period than the experiments shown in Figure 6.

We compared our results to an approach in which the trainable weights are updated based on an estimate of the noise, instead of using the real noise, similar to the EH rule described in Legenstein et al. (2010). The noise is estimated as the difference between the neural input ***a***, and the expected neural input $\bar{\text{a}},z_{\text{estim}}=\text{a}-\bar{\text{a}}$. The expected neural input $\bar{\text{a}}$ is simply an exponentially weighted moving average of ***a*** with a smoothing factor of 0.8. However, we found that this approach performed severely worse on the tasks we considered. A typical example of a 300 neuron network^2^ trained on the 3-bit decoder task is shown in Figure 7. The top panel plots the evolution of the reward during the training. It shows that not only is training much slower but also goes through a couple of bifurcations from which it is eventually unable to recover. The bottom panel shows why it is difficult to train the networks using this rule. We see that the noise estimation error slowly increases as the training continues. For this learning rule to work well, we require a good estimate of the noise throughout the entire learning process.

**Figure 8:**
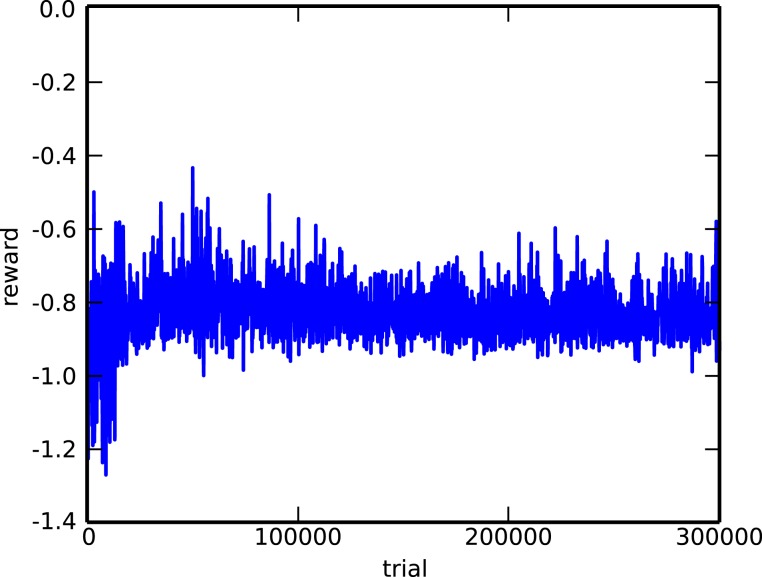
**Evolution of the reward for the delayed XOR task, using CMA-ES**. The task was simplified by removing all forms of stochasticity.

As a second comparison, we evaluated the performance of the covariance matrix adaptation evolution strategy (CMA-ES) (Hansen and Ostermeier, 2001), one of the most popular evolutionary algorithms. The algorithm is used on the 2-bit delayed XOR task. To make the task a bit simpler, we removed all sources of stochasticity (noise, initial neuron state), but apart from this, the setup is completely identical (identical network architecture, same initialization). The objective function to be maximized is the average reward across the four different possible inputs. Figure 8 shows the evolution of the reward during the first 300,000 trials. Comparing this to left panel of Figure 6, we can see that, although the RMH rule is able to find a good solution after 300,000 trials, CMA-ES is still nowhere near converging to a solution. The likely explanation for this is that, because the search space is so huge (about 10,000 dimensions), sample-based approaches like this one require an unfeasible number of samples.

**Figure 9:**
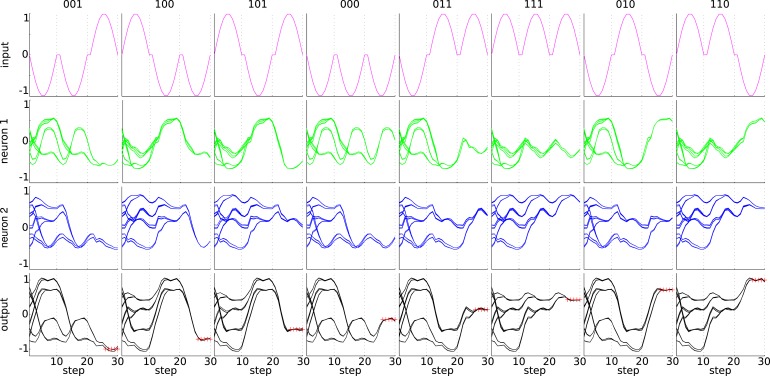
**State trajectories for the 3-bit classification task**. The top row shows the eight input sequences. The next two rows show the state trajectories of the two output neurons, which are summed to compute the observations for the given input sequence of the top row. The bottom row shows the observations (the sum of the two middle rows). The desired observation at the end of the trial is indicated by red crosses. The plots were generated by overlaying 50 random orderings of the input sequences.

Figure 9 shows in more detail what the embodied network of task 2 has learned, by overlaying the network output during 50 random orderings of the input sequences. Note that the classification result must only be available at the output during 5 time steps at the end of each trail (indicated by red crosses in the figure). As trails follow each other continuously, the variability of the state trajectories is due to the different initial states. Clearly, the network has learned the correct time window, as the classification result is available at the right time and only depends on the two previous bits and not on older inputs. We can identify a number of separate trajectories that keep track of the possible outcomes. It can be seen that within a trial, each additional bit that is offered at the input reduces the number of possible states of the system by half. Interestingly, the two neurons that generate the observations have different state trajectories, because the learning rule only quantifies the performance based on the observations, without directly enforcing a specific behavior of the neurons responsible for the observations.

**Figure 10:**
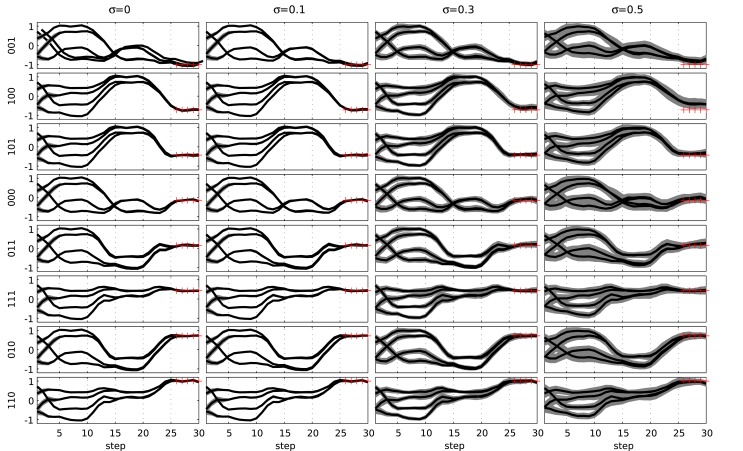
**Evaluation of a network trained for the 3-bit decoder task under the influence of input noise**. The noise amplitude increased from left to right. The eight possible inputs are shown from top to bottom. The gray area indicates 1 SD around the observations for each of the different state trajectories (thick lines). The red crosses indicate the target observations at the end of the trial.

Although the network was trained without any noise on the input signals, the resulting behavior is robust against such noise. In Figure 10, we show the behavior of the network when input noise is present. This plot was generated by first applying k-means clustering on the trajectories and then estimating the variance of each centroid. Shown are the various centroids and the SD of each. We see that the network is robust against high amounts of noise on the input data (*σ* up to 0.5), as the original trajectories are maintained.

**Figure 11:**
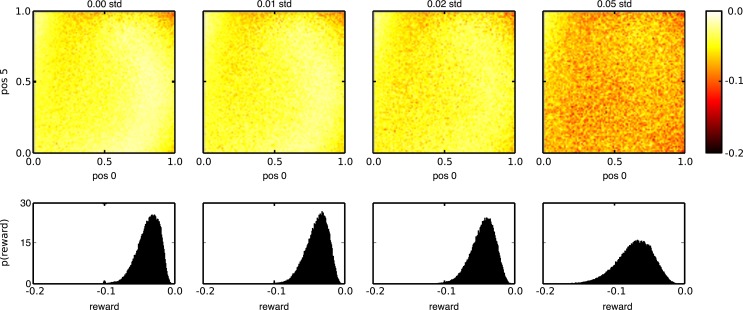
**Reward distribution for task 3 during testing with different state noise levels**. (Top) average reward (negative mean absolute error) for the whole range of input combinations. The horizontal and vertical axes of each plot indicate the initial and final values of the linear segments that need to be reproduced, in reverse order, at the output. (Bottom) sample distribution of the rewards for all inputs. The amount of injected state noise increases from left to right.

The same noise robustness can be observed on the last and most elaborate task. Figure 11 visualizes the rewards during testing for various state noise levels, using 100,000 random input trials per noise level. The noise was added to the internal neurons of the network, but not to the three neurons, which generate the observations. Each graph in the top panel shows the average rewards of the trained networks, across the whole spectrum of possible input sequences for a given level of input noise (increasing from left to right). The bottom panel shows the reward distribution, averaged across the different input trials, for each noise level. We see that without noise, the average reward remains close to its optimal value of 0 for most input patterns, although some regions of the input pattern space seem to be slightly more difficult. This demonstrates that the learning rule also works, although less perfectly than in the previous cases, when the relation between the internal states in the network and the way they are translated into actions and rewards is highly non-linear and when the input patterns do not fall into discrete categories. As noise levels increase, the average reward decreases, but only slightly, again displaying the noise robustness of the trained networks.

Finally, we show the virtue of the decorrelation learning rule [equation (4)] by slightly modifying the setup of the 3-bit decoder task. Instead of using uncorrelated Gaussian noise with SD *σ* = 0.05 for exploration, we generate the noise by sampling from a Gaussian distribution with SD *σ* = 0.035. The mean of the distribution is in turn sampled from a Gaussian with identical SD and zero mean, but only once per trial. During a single trial, the mean noise value is kept constant. This sampling procedure is nearly equivalent to the original one, except for the fact that two samples within the same trial are now highly correlated.

**Figure 12:**
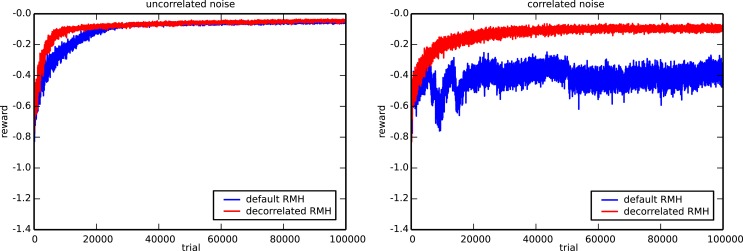
**Comparison of the default and the decorrelated RMH learning rules**. The evolution of the average reward for the 3-bit decoder task is shown in the presence of uncorrelated (left) and correlated (right) noise. The plot is generated using ten runs with different random initializations and input sequences.

Figure 12 compares the default and the decorrelation learning rules by plotting the average reward evolution for both the original task 2 setup and the setup with correlated noise. Again, we performed 10 runs with different random initializations and input sequences to generate each curve. In the left panel, we observe that in the context of uncorrelated noise, both learning rules give virtually identical results. In the case of correlated exploration noise though, the decorrelation learning rule does much better than the default one. The default RMH rule is able to learn at the start of a run, but quickly falls back and eventually settles on a suboptimal result. In contrast to this, the decorrelating version of the learning rule exhibits a very healthy learning curve and is only slightly affected by the fact that the exploratory noise is correlated.

### 4.2 Tensegrity experiments

Having shown the applicability of reward-modulated Hebbian learning on different tasks, using diverse setups, we now move on the tensegrity robot experiment. In this experiment, only a set of feedback weights are trainable, i.e., all neurons in a neural network controller have been replaced by the robot body.

**Figure 13:**
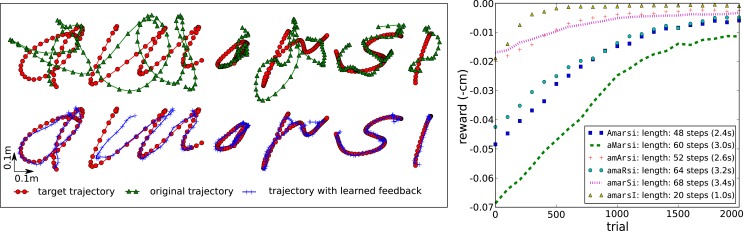
**Writing characters with a tensegrity end-effector**. (Top left) characters drawn with only the kinematic feed forward controller active. (Bottom left) characters drawn with the kinematic feed forward controller and the learned feedback controller active. (Right) learning curves for the different characters. The legend indicates the length of a trial.

The left panel of Figure 13 shows how the tensegrity robot performs when drawing characters between 20 and 68 time steps long (1–3.4 s). A different set of feedback weights was learned for each character; therefore, it is easy to predict the expected reward. To clarify, we estimated the expected reward for each character individually by averaging the rewards obtained during the previous 30 trials. As can be seen from the top row, the initial performance of the system with only the kinematic controller is very low, whereas the combination of both controllers, using our RMH learning rule [equation (5)], performs considerably better.

The plot on the right of Figure 13 shows the learning curves for each character, indicating that for most characters good results were obtained after 1000–1500 trials, which would be equivalent to <1 h real robot time for most characters. It is possible to accelerate learning by further tuning the learning parameters. We used a conservative level of exploration noise (*σ* = 5 mm) and a learning rate *α* = 1, which consistently resulted in stable feedback controllers. The learning rule did not achieve the same final reward for all characters (e.g., the “m”). This is due to physical limitations enforced on the motor commands.

**Figure 14:**
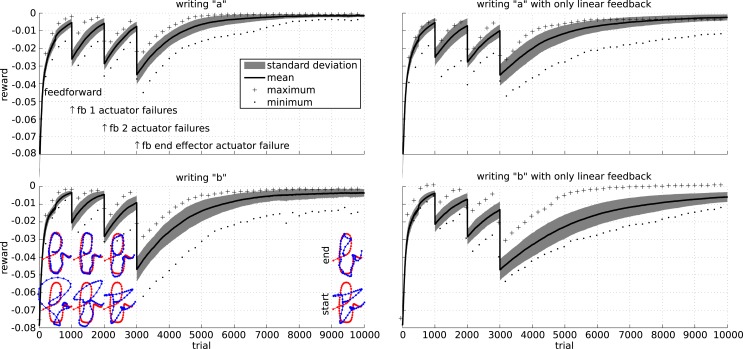
**Robustness of the learning rule for the writing task**. Initially, a feed forward controller is optimized. We then simulate a failure by making a single actuator follow its initial trajectory from trial 1000 onward. At the same time, the learning rule starts learning a set of feedback weights to compensate for the actuator failure. Similarly, we simulate a two actuator failure after trial 2000. At time 3000, we simulate a failure of an actuator directly attached to the end-effector. The top row shows the results for writing an “a” character, while the bottom row shows the results for a “b.” The left column show the results when the feedback includes the spring forces and the square of the spring forces, while the right column only includes the spring forces. The plots show the mean, maximum, minimum, and SD of the reward over 30 runs.

Finally, we simulate actuator failures. The results of these experiments are presented in Figure 14. As could be expected, the performance immediately drops significantly after each failure. By applying the RMH learning rule to the feedback controller, the system is able to recover from the various failures. To investigate the stability of the learning rule, each experiment was performed 30 times, with similar results.

## 5 Discussion

Hebbian theory is a well-established approach to explain synaptic plasticity between neurons. Over the years, many variations of the basic learning rule have been developed. Each of these had a specific application, ranging from unsupervised feature extraction to reinforcement learning. In this work, we handled Hebbian-like learning rules in which the synaptic plasticity is based on the correlation of the presynaptic neurons and an exploratory noise signal. The plasticity was modulated by a reward signal, resulting in a learning method that maximizes the expected reward of a trial.

We showed that this kind of learning can be applied outside the scope of traditional neural networks, namely in embodied computation. While similar rules have already been presented, we focused on reward learning in constrained recurrent neural networks and compliant robots. The rationale for this is our belief that both can be seen as computational resources and can therefore benefit from similar learning techniques.

Our work builds upon Legenstein’s (Legenstein et al., 2010), who considered simulated motor control tasks in combination with an instantaneous reward signal in an initially chaotic neural network. One significant difference with respect to our experimental setup is that Legenstein estimated the exploration noise as well as the expected reward. This allows for uncontrolled or unknown noise sources to be used, which adds to the biological plausibility of the method learning (Faisal et al., 2008). Covariance and noise-based rules have a strong biological foundation (Loewenstein and Seung, 2006; Soltani and Wang, 2006; Loewenstein, 2008). For example, it is well-known that neural networks in biology have intrinsic noise sources (Faisal et al., 2008), which could be used for learning (Maass, 2014). While this type of noise can sometimes be measured by external means (e.g., voltage clamps), a plasticity rule within the biological substrate cannot generally observe the noise signals, hence the importance of the noise estimator in Legenstein’s rule. In this work, we considered this approach briefly, but observed unfavorable results. The noise estimation scheme used by Legenstein requires the input and network dynamics to be temporally stable on small time scales, which likely explains our observations. However, apart from its biological plausibility, such a scheme is unnecessary in our context, as significant uncontrolled noise sources seem unlikely in robotics. Finally, we remark that Legenstein’s learning rule extends various earlier techniques with similar mathematical formulations (Fiete and Seung, 2006; Loewenstein and Seung, 2006; Loewenstein, 2008).

Another important difference of the learning rule that we considered with more biologically plausible alternatives, like the ones presented in Legenstein et al. (2008) and Soltoggio and Steil (2013), is that we employed trial-based learning. The fact that rewards are always distributed at the end of a learning episode in our setup allowed us to accumulate covariances throughout the episode, instead of making use of eligibility traces to keep track of the covariances in the recent past. This, in fact, makes it easier to solve the distal reward problem, since credit can only be assigned to exploration that happened during the same trial that the reward was received.

Our study of RMH learning in neural networks shows that the considered learning rule works for a wide range of conditions (trainability and observation functions) and for very different tasks. In addition, we have interpreted the network configuration of task 2, in which an entire subnetwork was not trainable, as a model for partially embodied computation. An interesting question in this case is whether learning in the trainable part of the neural network was fundamentally necessary in order to recognize the input patterns, or, in contrast, whether the necessary computations were already present in the network dynamics. In this case, as in a traditional reservoir computing setup, the learning only needed to provide a suitable mapping from the internal dynamics to the observation function.

The presented results show that after training, the system state evolves along a fixed number of highly robust trajectories. This phenomenon is not commonly observed in reservoirs without trained feedback weights, indicating that training half of the network at least provides feedback loops in addition to a suitable observation function. However, whether an actual trainable neural network has added value on top of these functionalities is not clear from the current experimental results. A more detailed analysis of the learning outcomes in assemblies of fixed and trainable substrates, as a model for partially embodied computation, is the subject of ongoing work.

Given the promising results we obtained in our simulations and the limited number of assumptions we had to make to obtain successful results, this work paves the way toward more complex control hierarchies for robot motor control, in which each level refines the output of the previous one. In this context too, our results raise some interesting questions, for instance, about the exact role of the very poorly performing kinematic controller in our experiments. In fact, the main goal of the kinematic controller is not to have the optimal performance, but rather to inject energy into the structure. In our previous work, we showed an example with an instantaneous reward function in which we first trained a feedback controller with known target signals using recursive least squares, and then proceeded to learn additional feedback signals using a reward-modulated Hebbian rule (Caluwaerts et al., 2012, section 5.1.3). The reason why an additional energy source is employed in both cases, is that it is hard to consistently learn pure feedback controllers with simple Hebbian-like learning rules. A small change in a feedback weight can cause the system dynamics to fade out, which often results in instability. Therefore, an easy and efficient solution is to use an additional input that consistently pumps energy into the system. In principle, this can be accomplished using a feedback controller as in our previous work, a simple feed forward controller as we use here, or another controller, such as a central pattern generator. More extensive research is needed to determine how much of the workload can be offloaded to the lowest level, partially embodied feedback controller and how this scales to more complex control tasks, e.g., involving more complex robot bodies.

In summary, our main conclusion is that reward-modulated Hebbian plasticity provides a simple, yet effective tool for bridging learning in recurrent neural networks and the exploitation of the own dynamics of compliant robots. This strengthens our belief that both the body and the neural network can be used as computational tools and that they should be combined in a self-organizing way into partially embodied hierarchical controllers.

## Author Contributions

JB and KC performed the experiments. All authors contributed to the interpretation and structuring of the results and to writing the paper.

## Conflict of Interest Statement

The authors declare that the research was conducted in the absence of any commercial or financial relationships that could be construed as a potential conflict of interest.

## Footnotes

^1^We use the notation *x* to denote a scalar, ***x*** for a vector, and ***X*** for a matrix. In general ***x***_i_ is the *i*th row of ***X*** and *x_i_* is the *i*th element of ***x***.

^2^The experimental results using only 100 neurons are qualitatively similar, but less pronounced.

## Appendix

### A Stabilized Reward-Modulated Hebbian Rule

Oja’s rule (Oja, 1982) and its extension, the generalized Hebbian algorithm or Sanger’s rule (Sanger, 1989), provide a single layer neural network implementation to compute principal components. Contrary to pure Hebbian plasticity, the learning rules are stable, because they force the norm of the weight vectors to unity. Unlike in the unsupervised learning case, reward-modulated rules tend to be stable in practice (i.e., the trained weights remain bounded). However, it can still be useful to control the norm of the weights as this can have practical implications. For example, in a robotics application, this would allow for limiting the required feedback gain and thus the required motor power. From a theoretical point of view, it is also instructive to see how the learning rules employed throughout this paper resemble the now classic rule discovered by Sanger over 20 years ago. In this section, we provide a similar derivation for the RMH learning rule that we studied in this work.

To simplify the notation, we start by defining a number of variables:

(A1) $$\text{E}={\text{Z}}^{T}\text{X}$$

(A2) $$r\prime =r-\bar{r}.$$

The basic learning rule we studied can now be written in element-wise form as:

(A3) $$w_{\mathrm{ij}}\left[n+1\right]=w_{\mathrm{ij}}\left[n\right]+\alpha r\prime e_{\mathrm{ij}}$$

Oja’s rule is a first order approximation to the normalization of the weights at every update step:

(A4) $$w_{\mathrm{ij}}\left[n+1\right]=b_{i}\frac{w_{\mathrm{ij}}\left[n\right]+\alpha r\prime e_{\mathrm{ij}}}{∥\;{\text{w}}_{i}\left[n\right]+\alpha r\prime {\text{e}}_{i}\;∥},$$

where ***b*** contains the desired *L*_2_ norms of the weight vectors.

We now consider the linearization of this rule for small learning rates *α*. To further simplify the notation, we drop the time index and consider a single output dimension:

(A5) $$w_{j}\approx b\frac{w_{j}+\alpha re_{j}}{∥\;\text{w}+\alpha r\text{e}\;∥}{\left|\right.}_{\alpha =0}+\alpha b\left(\frac{\partial }{\partial \alpha }\frac{w_{j}+\alpha re_{j}}{∥\;\text{w}+\alpha r\text{e}\;∥}\right)\;{\left|\right.}_{\alpha =0}$$

A straightforward calculation shows that the part inside the parentheses of the second term can be written as:

(A6) $$\frac{\partial }{\partial \alpha }\frac{w_{j}+\alpha re_{j}}{∥\text{w}+\alpha r\text{e}∥}{\left|\right.}_{\alpha =0,∥\text{w}∥=b}=\frac{re_{j}\text{w}+\alpha r\text{e}∥\;-\left(w_{j}+\alpha re_{j}\right)\left(\text{w}+\alpha r\text{e}\right)\cdot \left(r\text{e}\right)}{∥\;\;\text{w}+\alpha r\text{e}{∥}^{2}}{\left|\right.}_{\alpha =0,∥\text{w}∥=b}$$

(A7) $$\;=\frac{1}{b}re_{j}-\left(\frac{r\text{w}\cdot \text{e}}{b^{3}}\right)w_{j},$$

assuming ∥***w*** ∥ = *b* and *α* = 0.

The complete learning rule can therefore be written as:

(A8) $$w_{j}=b\frac{w_{j}}{b}+\alpha b\left(\frac{1}{b}re_{j}-\left(\frac{r\text{w}\cdot \text{e}}{b^{3}}\right)w_{j}\right)$$

(A9) $$=w_{j}+\alpha r\left(e_{i}-\frac{w_{j}}{b^{2}}\text{w}\cdot \text{e}\right)\;.$$

The matrix form of the rule for multiple outputs is given by:

(A10) $$\Delta \text{W}=\alpha r\prime \left(\text{E}-{\mathrm{diag}}_{v}\left(\text{b}\right)-2{\mathrm{diag}}_{v}\left({\mathrm{diag}}_{m}\left({\text{EW}}^{T}\right)\right)\text{W}\right),$$

where the diag_v_ operator transforms a vector into a diagonal matrix, and the diag_m_ operator transforms a matrix into a vector, containing its diagonal elements. The time complexity to this rule is of the order O(*mn*), with *m* the number of outputs and *n* the number of inputs. The resulting stabilized learning rule very closely resembles Sanger’s rule.
